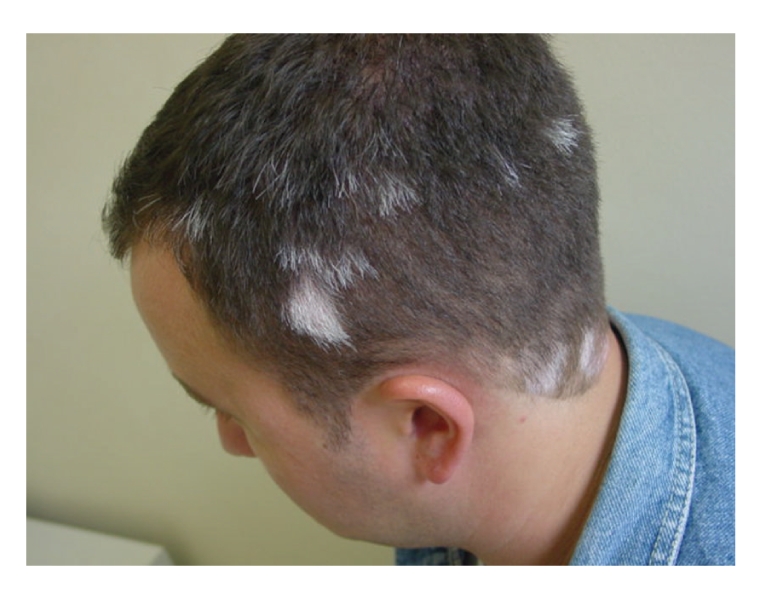# Vitiligo after Diphencyprone for Alopecia Areata

**DOI:** 10.1155/2010/171265

**Published:** 2010-05-11

**Authors:** Mario Cezar Pires, João Mauricio Martins, F. Montealegre, Flávia Romero Gatti

**Affiliations:** ^1^Dermatology Department, Complexo Hospitalar Padre Bento de Guarulhos, Saõ Paulo, Brazil; ^2^Ponce School of Medicine, Puerto Rico, USA

## Abstract

The topical immunotherapy is used to treat alopecia areata and recalcitrant warts since the 1970s. Diphencyprone is a contact sensitizer used to treat dermatological conditions resulting from as altered immunological state, such as extensive alopecia areata, being partially effective and safe. Side effects include local eczema with blistering, regional lymphadenopathy and contact urticaria. Rare adverse effects include an erythema multiforme-like reaction, hyperpigmenttion, hypopigmentation, and vitiligo. We report a 30-year-old, Brazilian male who developed vitiligo lesions following DPCP therapy for alopecia areata.

## 1. Introduction

Topical immunotherapy is used to treat alopecia areata and recalcitrant warts since the 1970s [1]. Daman, Rosenber, and Drake in 1978, demonstrated the effectiveness of dinitrochlorobenzene (DNCB) in 2 patients with Alopecia Areata [2], and new studies have been conducted in the attempt to establish the therapeutic potential of this agent [3–5]. However, some authors questioned the safety of this chemical, suggesting a possible carcinogenic potential [6–8]. To this end, mutagenic properties of DNCB have been showed in vitro studies of *Salmonella enteritidis *serotype *typhimurium *[9]. Therefore, new substances with the same properties of DNCB have been introduced in clinical practice, and these include dibutil-ester-squaric acid (DESA) and diphenylcyclopropenone acetate (diphencyprone—DPCP) [1, 9]. Diphencyprone is a contact sensitiser used to treat dermatological conditions resulting from as altered immunological state, such as extensive alopecia areata [10–13]. Happle et al. in 1983 used, for the first time, diphencyprone in 27 alopecia areata patients [10]. Their results were exciting, with 67% of hair regrowth. Side effects include local eczema with blistering, regional lymphadenopathy and contact urticaria. Rare adverse effects include an erythema multiforme-like reaction, hyperpigmenttion, hypopigmentation, and vitiligo [11, 12]. Recently, Pan et al. reported 4 patients with alopecia areata who developed the side effect of vitiligo following DPCP immunotherapy [13]. Herein we report an additional case of vitiligo following DPCP therapy for alopecia areata. The patient was a 30-year-old, Brazilian male, white, salesman with one-year history of hair loss. The diagnosis of alopecia areata was made and more lesions appeared. The dermatological exam showed rounded, regular patches of alopecia varying from 1 to 2 cm, mostly in occipital and parietal regions. He did not present scales or atrophy in the affected areas. There were alopecia patches in beard and eyelashes areas as well. The general physical examination was normal. The patient did not have personal and familial history for vitiligo, atopy or other autoimmune diseases.

The laboratory exams, including thyroid auto-antibodies, ANA, CBC and biochemical were normal.

Oral prednisone 40 mg per day was prescribed for 3 weeks. After this period, the hair loss had stopped, but the alopecia patches remained. The patient had been started on topical immunotherapy with diphencyprone, rigorously following a pre-established protocol [11]. The typical concentration of DPCP used was 0.02%, after the prior sensitization. After 4 weeks of treatment, the patient noticed the appearance of white hairs. 5 months later there was complete recovery of the alopecia areata, however, with white hairs and vitiligo-like hypopigmented lesions in the same areas (Figure 1). The treatment was interrupted and after 3 months the hypopigmented lesions did not develop pigmentation. The patch test that was performed with DPCP 0.02% was applied in the left arm and a positive reaction was observed after 48 hours and 96 hours. After 30 days, we observed a hypopigmented lesion at the patch test site. The patient did not recover the pigmentation after desonide lotion.

## 2. Discussion

Some authors have described a correlation between vitiligo and alopecia areata, ranging from 4 to 9% [11, 12]. Our patient did not have personal or familial evidences of vitiligo; it is not possible to prove that the hypopigmented patches were a primary form of the disease or a consequence of the DPCP treatment. However, the reproduction of the hypopigmentation after the patch test with DPCP is highly suggestive of a therapy complication. We did not find other report where the vitiligo lesion DPCP induced was reproduced by patch test. The pathogenesis of this phenomenon is not known but it has been postulated that the contact sensitiser may have induced vitiligo through a direct cytotoxic effect on the melanocytes following systemic absorption. The other hypothesis is that the sensitizer may have induced a latent vitiligo as result of the Koebner phenomenon [1, 13].

Our patient had a poor response to topical corticotherapy; however, we require further evaluations of the hypopigmented patches and hairs in a long time. In most of the cases, repigmentation may occur with treatment such as phototherapy, but complete recovery is uncommon, like Pan et al. showed [13]. The true incidence of vitiligo in patients using DPCD is not defined; however, it is imperious to explain this possibility to the patients who have a personal or familial vitiligo history. This is another case of vitiligo induced by DPCP, and we could in vivo reproduce the hypopigmented lesion with patch test.

## Figures and Tables

**Figure 1:**